# Supplementary material for: Widespread bacterial lysine degradation proceeding via glutarate and L-2-hydroxyglutarate
Source: Nat Commun. 2018 Nov 29;9:5071. doi: 10.1038/s41467-018-07563-6 (PMC6265302; doi:10.1038/s41467-018-07563-6)
Supplement: Supplementary file 1 — Supplementary Information [file 41467_2018_7563_MOESM1_ESM.pdf]

Supplementary Information for Knorr *et. al*:

Widespread bacterial lysine degradation proceeding via glutarate and *L*-2-hydroxyglutarate

Supplementary Information

Widespread bacterial lysine degradation proceeding via glutarate and *L*-2-hydroxyglutarate

Sebastian Knorr[1,2*], Malte Sinn[1,2*], Dmitry Galetskiy[1], Rhys M. Williams[3], Changhao Wang[1], Nicolai Müller[3], Olga Mayans[2,3], David Schleheck[2,3], & Jörg S. Hartig[1,2]

1: Department of Chemistry, University of Konstanz

2: Konstanz Research School Chemical Biology (KoRS-CB)

3: Department of Biology, University of Konstanz

Content:

Supplementary Figure 1: ^{1}H NMR spectra of α-ketoglutarate and glutarate.

Supplementary Figure 2: ^{1}H NMR spectra of the products of the CsiD reaction.

Supplementary Figure 3: Stereospecificity of the CsiD reaction.

Supplementary Figure 4: Phylogenetic distribution of CsiD.

Supplementary Figure 5: Inhibitors of glutarate hydroxylase CsiD.

Supplementary Figure 6: Ligand complexation by CsiD.

Supplementary Figure 7: Ubiquinone-1 reduction by purified LhgO

Supplementary Figure 8: LC/MS chromatogram of GabT/D enzyme reaction products.

Supplementary Figure 9: LC/MS chromatograms of PatA/D enzyme reaction products.

Supplementary Figure 10: Growth analysis of *E. coli* knockout strains

Supplementary Figure 11: Coupled transamination and dehydrogenation by PuuE/Sad..

Supplementary Figure 12: Filter binding assay of CsiR.

Supplementary Figure 13: Hydroxyl radical footprinting of CsiR binding to the CsiD promoter region.

Supplementary Figure 14: SPR analysis of the interaction of CsiR with the CsiD promoter region.

Supplementary Figure 15: Growth curve of E. coli BW25113 compared to the ΔcsiD in LB medium.

Supplementary Table 1: X-ray data statistics and model refinement parameters

Supplementary Table 2: Ions for identification and quantification of compounds by LC-MS

Supplementary Table 3: Labelling pattern and quantification of metabolites measured via HPLC/MS of *E. coli* WT*,* Δ*csiD* and Δ*gabT*

Supplementary Table 4: List of primers used in the study

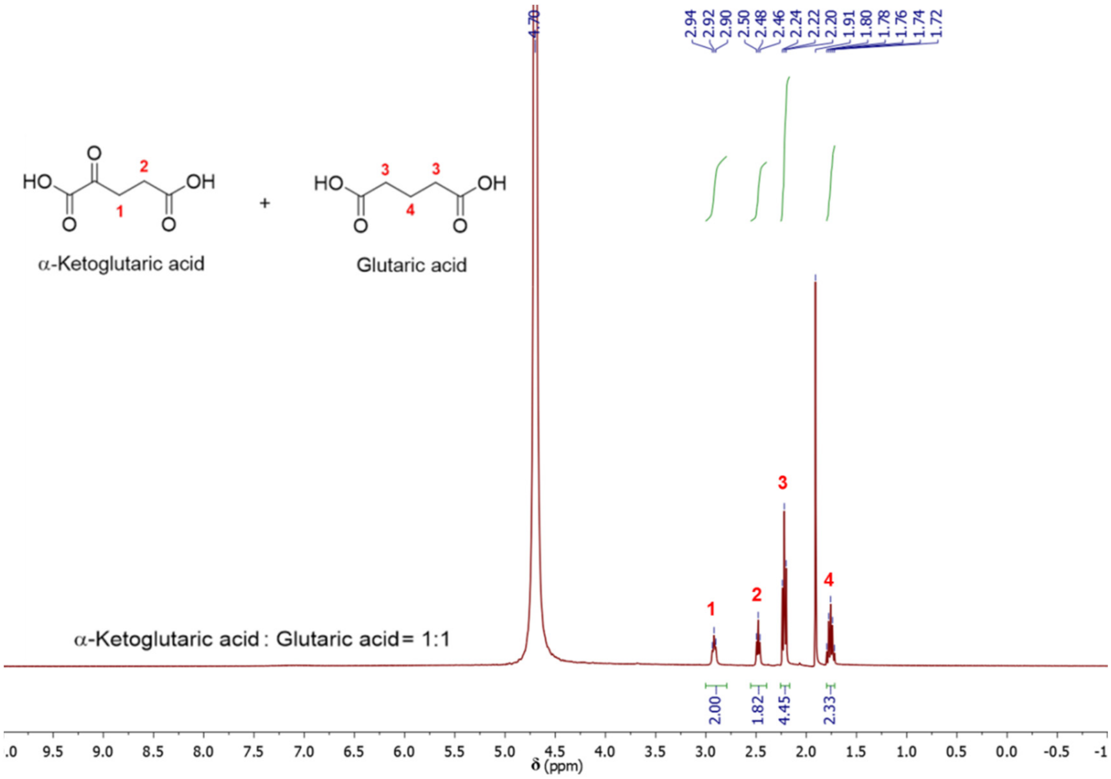

Supplementary Figure 1: ^{1}H NMR spectra of α-ketoglutarate and glutarate in ammonium acetate buffer.

^{1}H NMR (400 MHz, D_2O, α-ketoglutaric acid : glutaric acid = 1:1) 2.92 (t, J = 6.8 Hz, 2H, **1-H**), 2.48 (t, J = 6.9 Hz, 2H, **2-H**), 2.22 (t, J = 7.6 Hz, 4H, **3-H**), 1.76 (p, J = 7.6 Hz, 2H, **4-H**). Peak at 1.91 ppm arises from acetate.

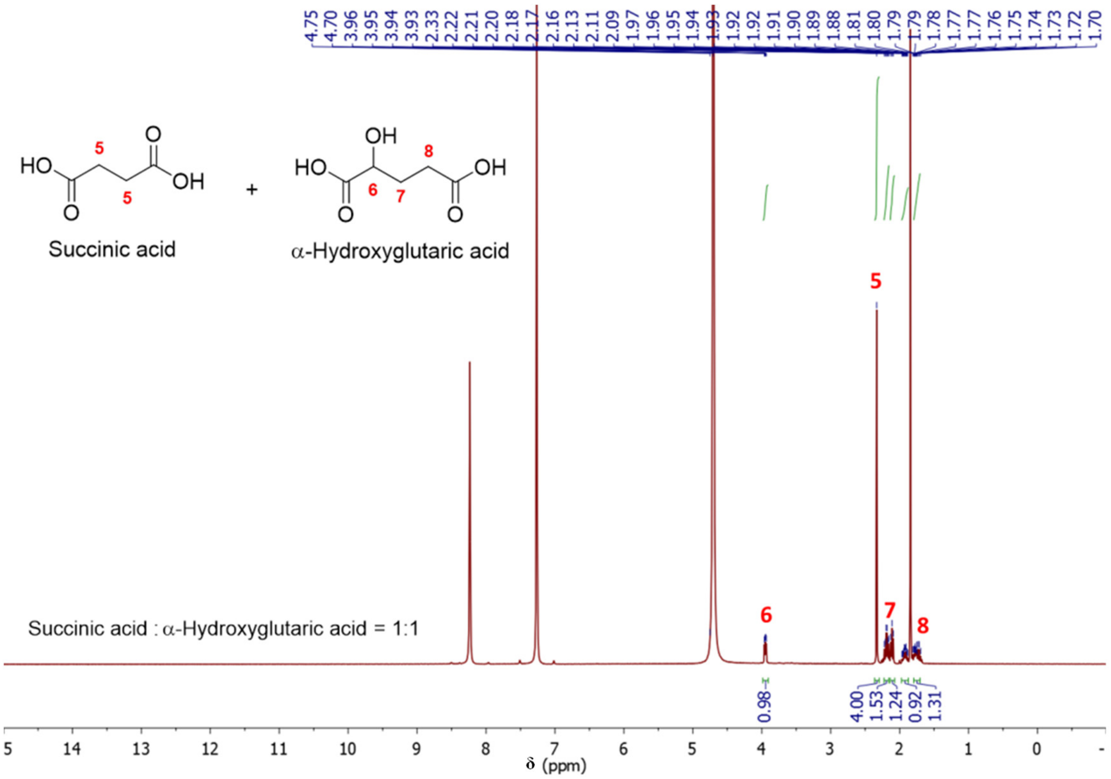

Supplementary Figure 2: ^{1}H NMR spectra of the products of the CsiD reaction in ammonium acetate buffer.

^{1}H NMR (400 MHz, D_2O, succinic acid : *L*-2-hydroxyglutarate = 1:1) δ 3.95 (dd, J = 7.6, 4.1 Hz, 1H, **6-H**), 2.33 (s, 4H, **5-H**), 2.19 (td, J = 9.6, 5.9 Hz, 1H, **7-H**), 2.11 (t, J = 7.7 Hz, 1H, **7-H**), 1.97 – 1.87 (m, 1H, **8-H**), 1.80 – 1.70 (m, 1H, **8-H**). Peak at 1.89 ppm arises from acetate. Peaks between 7-9 ppm arise from the enzyme.

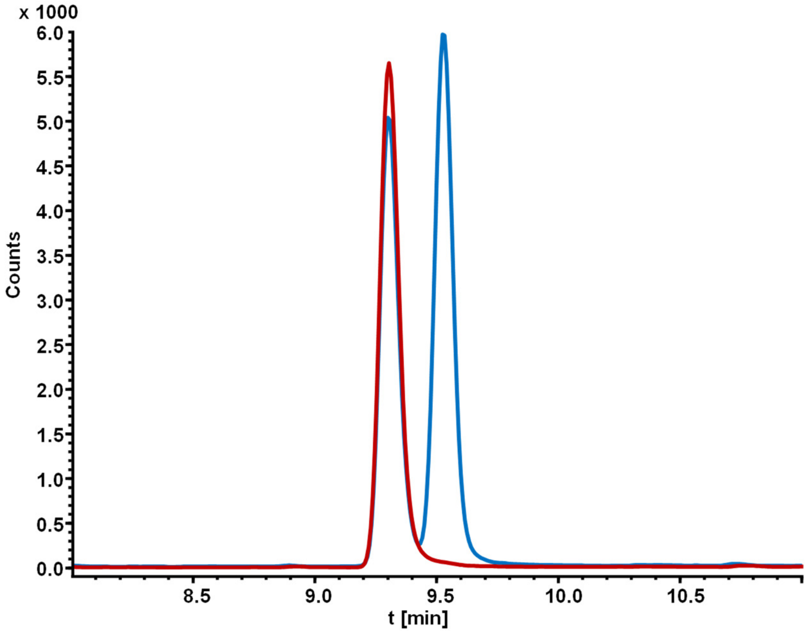

Supplementary Figure 3: Stereospecificity of the CsiD reaction. *L*-2-hydroxyglutarate produced by CsiD and in a mixture with D-2-hydroxyglutarate was derivatized with DATAN[1] and analyzed by LC-MS. Chromatograms of the derivatized reaction product (red) and the mixture (blue) were recorded in negative SIM at a *m/z* ratio of 363. The reaction product elutes as a single peak clearly separated from the second peak that represents derivatised *D*-2-hydroxyglutarate.

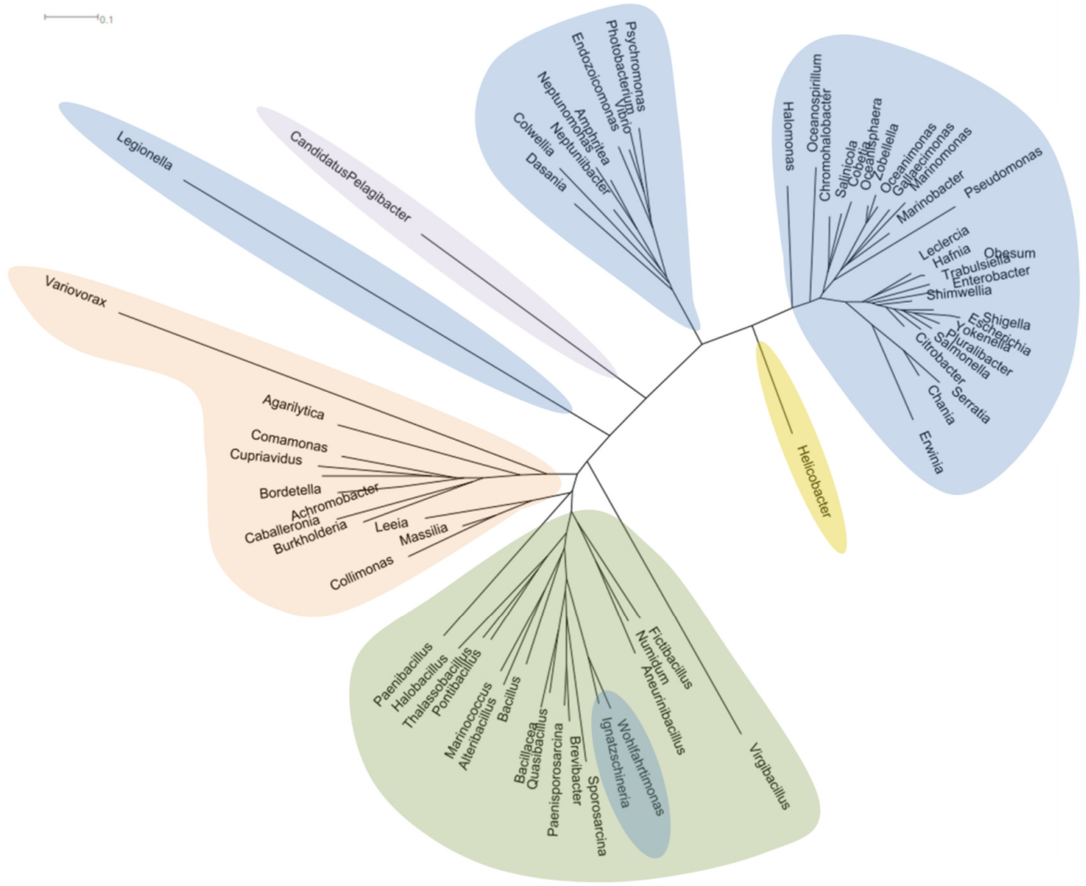

Supplementary Figure 4: Phylogenetic distribution of CsiD. A neighbourhood joining phylogeny of CsiD based on *E. coli* K-12 CsiD was built retaining sequences with more than 70% coverage and 25% identity. Homologs of *E. coli* CsiD were searched in the refseq database (last access May 2018)[2] using pBLAST. Bacterial classes are as indicated: α- (purple), β- (orange), □- (blue), and ε- (yellow) proteobacteria and bacilli (green).

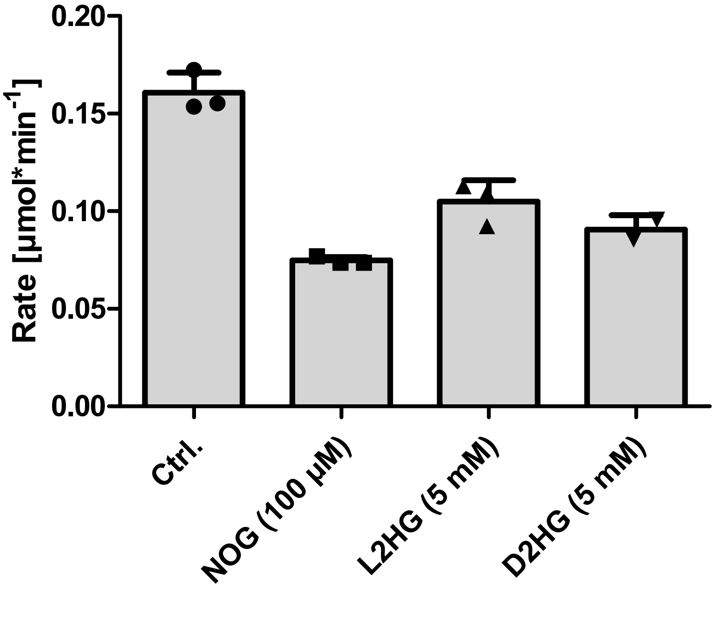

Supplementary Figure 5: Inhibitors of glutarate hydroxylase CsiD. Reaction rate was determined in a Clark-type oxygen electrode monitoring the oxygen consumption. The rate was determined in the presence of the αKG-dependent dioxygenase inhibitor N-oxalylglycin (NOG), L2HG, D2HG. In the control reaction (Ctrl.) no inhibitor was present. The reported K_i of L2HG for α-KG dependent dioxygenases is 628 +/- 0.036 µM, in contrast the estimated K_i of L2HG for CsiD is > 10 mM. The estimated K_i for NOG and D2HG match the reported K_i for αKG-dependent dioxygenases[3]. Data represent the mean of triplicate measurements with error bars representing standard deviations. (D2HG; n=2).

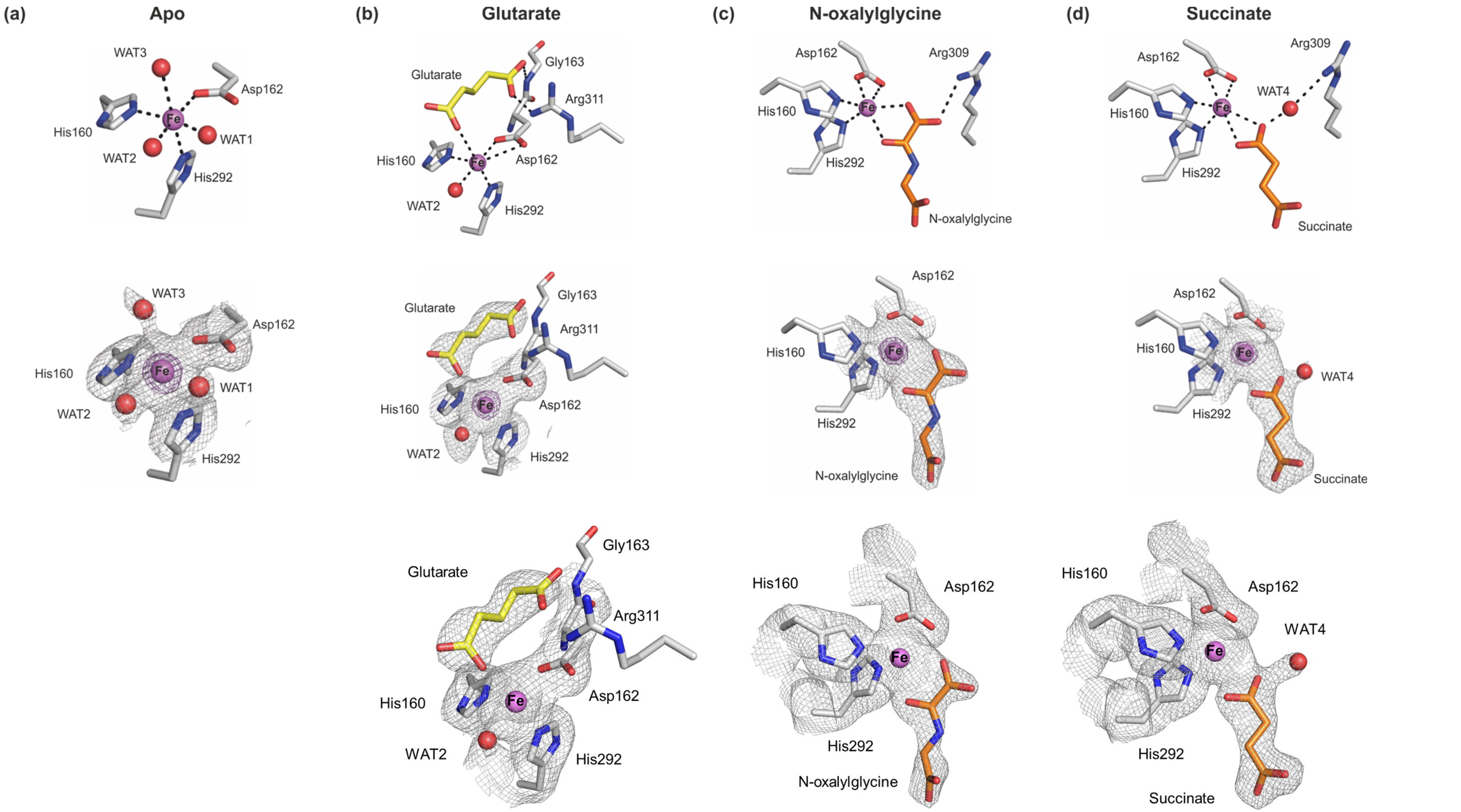

Extended Data Fig. 6: Ligand complexation by CsiD. Detailed view of CsiD in complex with its ligands. Upper panel shows interactions (dashed lines), middle panels display (2Fo-Fc)αc electron density maps contoured at 1σ (grey) and 7σ (purple). Lower panels show ligand complexes with (2Fo-Fc)αc electron density omit maps contoured at 0.9σ. For map calculation, ligands were removed from the models and the non-liganded models subjected to simulated annealing refinement (5000 K) in PHENIX.

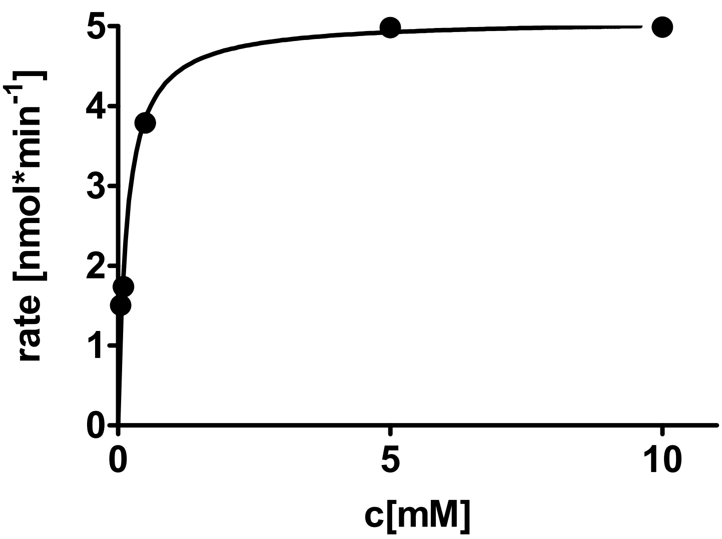

Supplementary Figure 7: Ubiquinone-1 (UQ_1) reduction by purified LhgO as a function of L2HG concentration. Purified LhgO reaction rate (R) was measured at different L2HG concentrations and is given as nmol UQ_1 reduced per time. Specific activity of LhgO was determined as 0.33 +/- 0.002 µmol/min*mg. LhgO showed a K_M of 162 µM for L2HG and a V_{max} of 5.1 nmol/min.

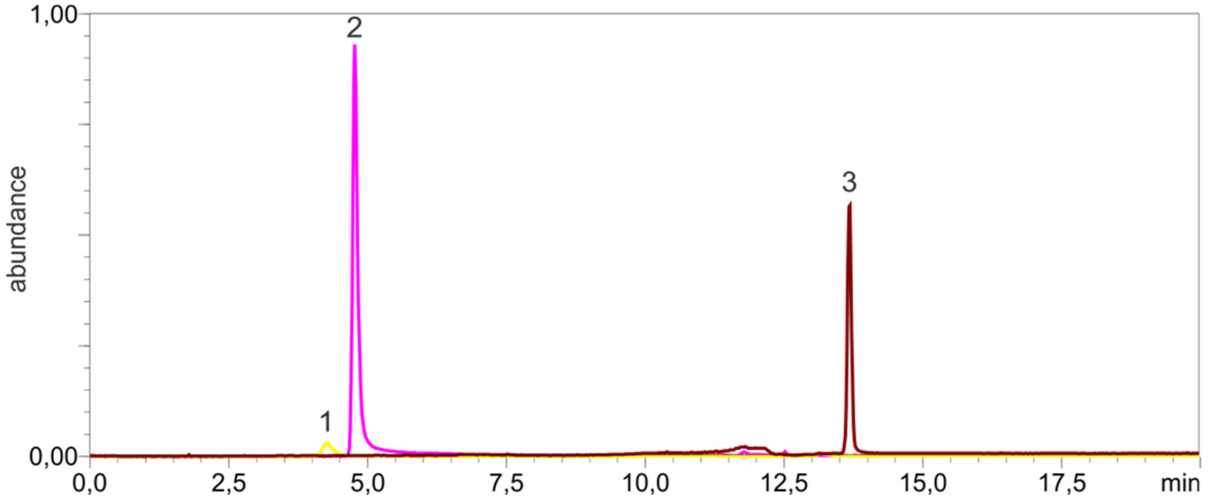

Supplementary Figure 8: LC/MS chromatogram of GabT/D enzyme reaction products.
1) Glutarate semialdehyde (*m/z* = 115.0; RT = 4.3 min; yellow). 2) Glutaric acid (*m/z* = 131.0; RT = 4.9 min; magenta. 3) Glutamic acid (*m/z* = 148.1; RT = 13.7 min; brown).

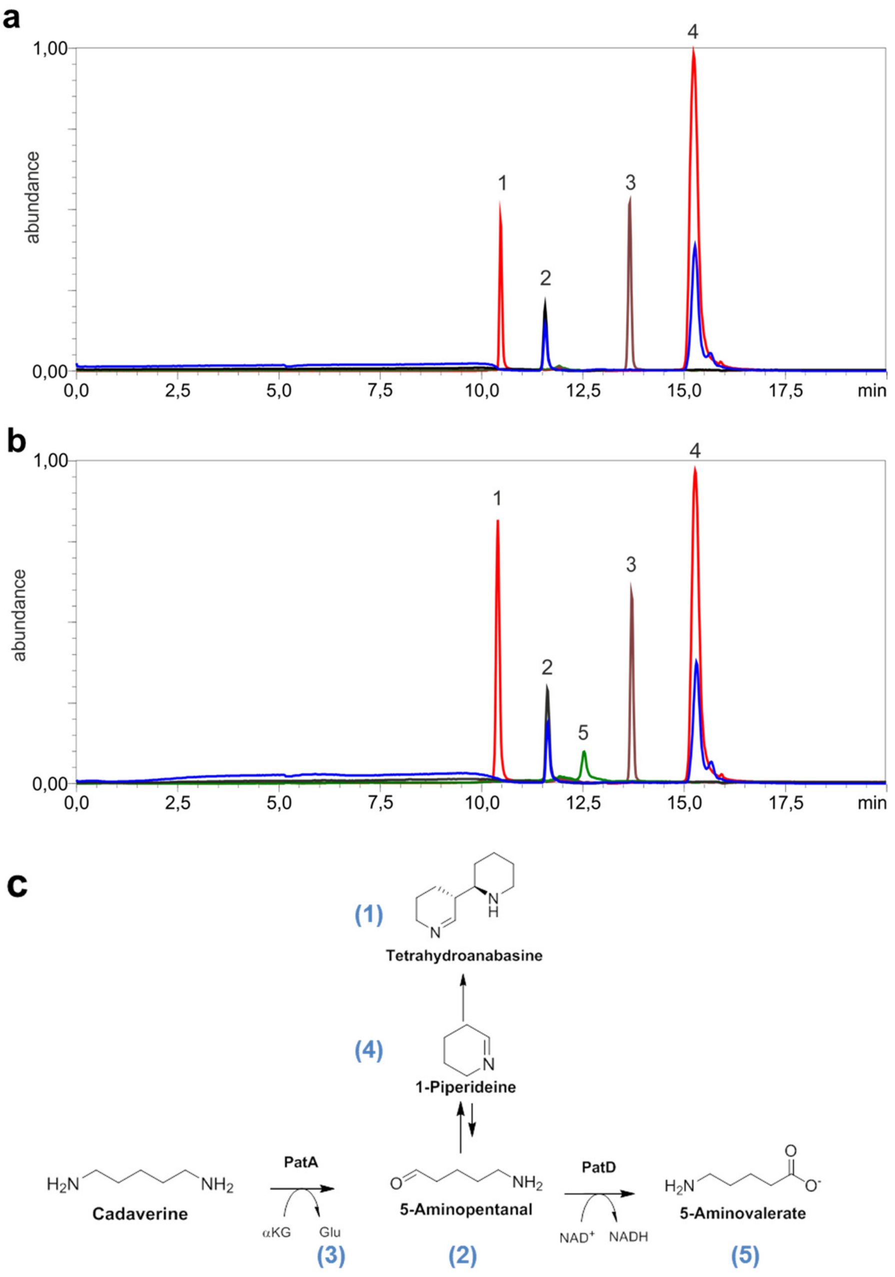

Supplementary Figure 9: LC/MS chromatograms of PatA/D enzyme reaction products. 1) Piperideine dimer (Tetrahydroanabasine) (*m/z*=167.2; RT=10.5 min; red). 2) 5-aminopentanal (*m/z*=102.1; RT=11.6 min; black. 3) glutamic acid (*m/z*=148.1; RT=13.7 min; brown); 4) 1-Piperideine (*m/z*=84.1; RT=15.3 min; blue); 5) 5-Aminovaleric acid (*m/z*=118.1; RT=12.5 min; green). **a,** PatA single reaction. PatA transfers the amino group of cadaverine to αKG, producing 5-aminopentanal (2) and glutamic acid (3). **b,** in a coupled PatA/D reaction 5-aminopentanal (2) is oxidised to 5-aminovaleric acid (5) by PatD. Aminopentanal cyclized spontaneously to 1-piperideine (4) during MS analysis (likely during ionisation), which spontaneously dimerises to yield tetrahydroanabasine. For this reason, 5-aminopentanal is detected at RT=11.6 min together with 1-piperideine. At RT=15.3 min the 1-piperideine monomer is detected together with its dimer. **c**, reactions catalyzed by PatA/D and spontaneous reactions of 5-aminopentanal.

		N-Source			C-Source			
	M9	Lysine	Cad	AVA	Lysine	Cad	AVA	GA
WT	+++	+	+	++	-	-	-	-
ΔpatA	+++	+	+	+	-	-	-	-
ΔpatD	+++	+	+	++	-	-	-	-
ΔgabP	+++	+	+	+	-	-	-	-
ΔgabT	+++	+	+	+	-	-	-	-
ΔcsiD	+++	+	+	+	-	-	-	-
ΔlhgO	+++	+	+	++	-	-	-	-
ΔcsiR	+++	+	+	++	-	-	-	-

Supplementary Figure 10: Growth analysis of *E. coli* knockout strains. *E. coli* strains were grown in minimal medium containing 10 mM of the respective nitrogen (N-) or carbon (C-) source. Growth was assessed by measuring OD_{600} and distinguished between good growth (dark green, +++), intermediate growth (light green, ++), low growth (orange, +) and no growth (red, -), see Methods for OD thresholds. Evaluated were the mean of independent triplicates.

E. coli is not able to grow on minimal medium containing the intermediates of the proposed pathway (lysine, Cad, AVA, GA) as carbon sources. *E. coli* wildtype, *ΔpatD*, *ΔlhgO* and *ΔcsiR* are able to grow to an intermediate level with AVA as a nitrogen source. If the importer GabP is knocked-out, growth on AVA is impaired as for the aminotransferases PatA and GabT. Additionally, *E. coli* shows a growth defect on AVA for *ΔgabP* and *ΔcsiD*.

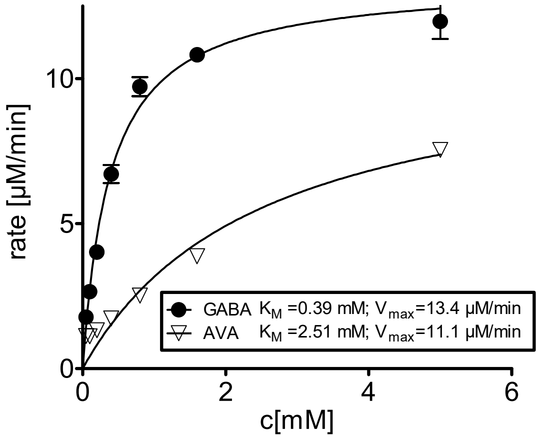

Supplementary Figure 11: Coupled transamination and dehydrogenation by PuuE/Sad. Coupled enzyme kinetics of AVA (triangles) by the putrescine transaminase PuuE and the succinate semialdehyde dehydrogenase Sad in comparison to the known substrate GABA (circles). Data is shown as mean of triplicate measurements with error bars representing standard deviations. Experimental procedure was conducted as described in materials and methods.

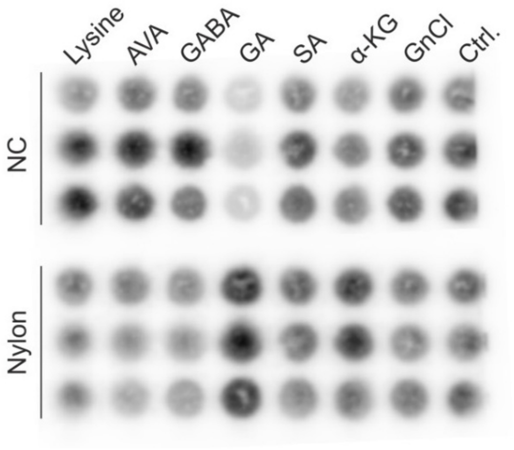

Supplementary Figure 12: Filter binding assay of CsiR. Ligand specificity of CsiR repression determined by a filter binding assay of CsiR and a dsDNA sequence derived from the *csiD* operon promoter region in presence of annotated compounds (1 mM each, GnCl: Guanidiniumchloride, Ctrl: no ligand). CsiR-bound radioactive DNA is retained on a nitrocellulose membrane. GA specifically reduces binding of CsiR to the DNA.

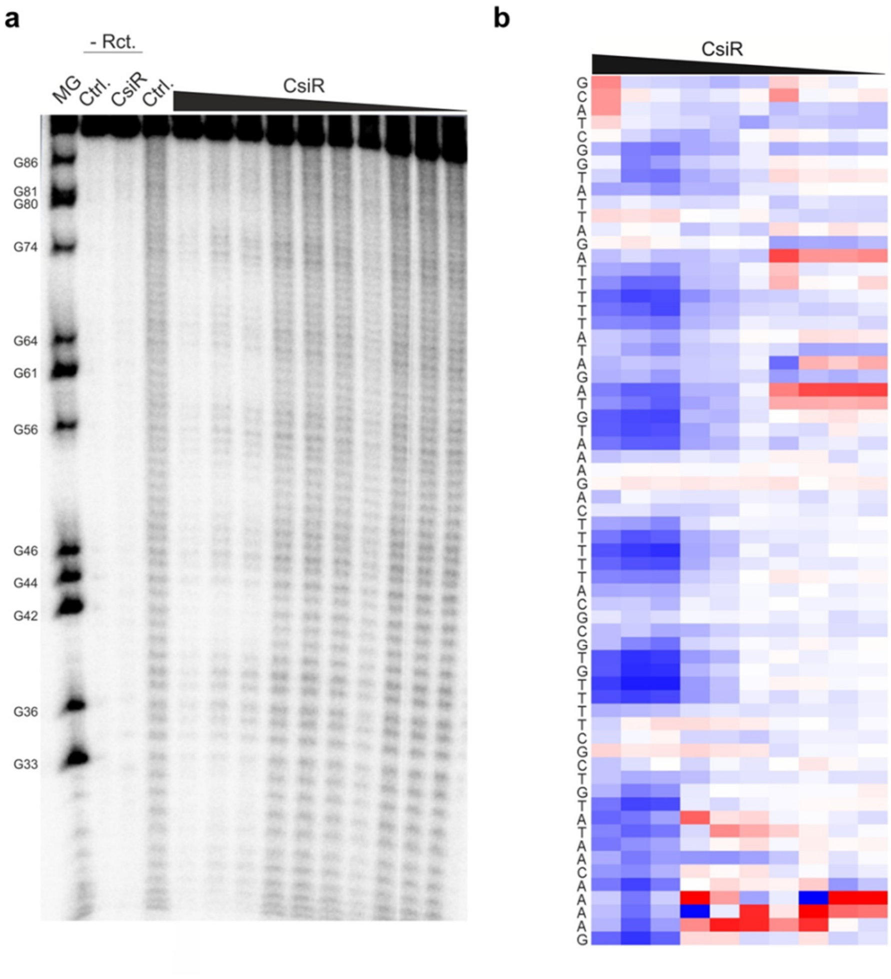

Supplementary Figure 13: Hydroxyl radical footprinting of CsiR binding to the CsiD promoter region. a Radiograph of the CsiR hydroxyl footprint. A G-specific Maxam-Gilbert (MG) reaction was performed to assign the resulting bands. For the non-reaction controls (-Rct.) no cleavage is observable. Cleavage of the DNA by hydroxyl radicals was conducted in the absence (Ctrl.) and presence of decreasing concentrations of CsiR. Regions of reduced cleavage can be observed in the presence of CsiR. **b** Analysis of the hydroxyl radical footprint with SAFA[4]. Each pixel represents a band of the radiograph and is aligned to the DNA sequence ranging from G22 to G86. Intensity was normalized to the control reaction (Ctrl.). Areas of protection show up in blue (less cleavage), whereas increased cleavage is depicted in red.

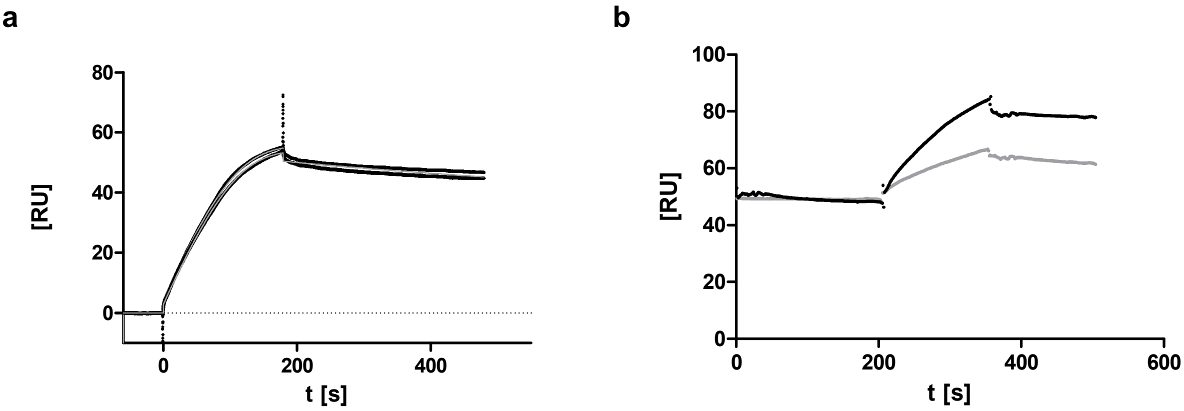

Supplementary Figure 14: SPR analysis of the interaction of CsiR with the CsiD promoter region. a, analysis of the binding kinetics of 0.9 µM CsiR to the CsiD operon. Two independent experiments (black) were fitted (grey) assuming a 1:1 interaction. **b,** interaction of CsiR to the CsiD promoter region is reduced in the presence of 1 mM glutarate (grey) compared to the control (black).

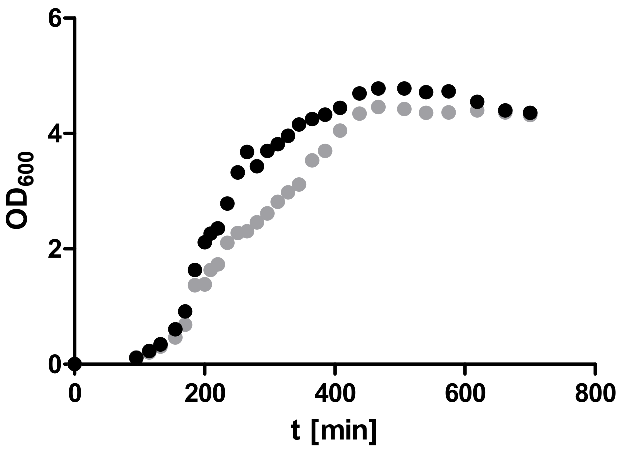

Supplementary Figure 15: Growth curve of *E. coli* BW25113 compared to the *ΔcsiD* strain in LB medium. Growth was assessed by recording the optical density at 600 nm. *ΔcsiD* (grey) exhibits a growth defect around $OD_{600} \approx 2$ compared to the wildtype strain. The shown curve is a representative of 5 individual experiments.

Supplementary Table 1: X-ray data statistics and model refinement parameters

	**CsiD APO**[a]	**CsiD GA**[a]	**CsiD NOG**[a]	**CsiD SA**[a]
PDB accession code	6GPE	6HL8	6GPN	6HL9
**Data collection**				
Space group	$P\ 4\ 2_1\ 2$	$P\ 4\ 2_1\ 2$	$P\ 4\ 2_1\ 2$	$P\ 4\ 2_1\ 2$
Cell dimensions				
a=b, c (Å)	121.20, 136.91	120.80, 136.42	121.38, 136.57	123.03, 138.33
Resolution (Å)	42.85 - 2.20 (2.25 - 2.20)[b]	42.56 - 2.40 (2.45 - 2.40)[b]	42.91 - 2.20 (2.24 - 2.20)[b]	46.11 - 2.30 (2.35 - 2.30)[b]
Unique reflections	47756 (3139)	40085 (2354)	52380 (2710)	47789 (2341)
Redundancy	15.6 (15.6)	7.26 (7.53)	22.98 (21.72)	26.7 (26.9)
Completeness (%)	91.5 (94.4)	99.9 (100.0)	99.99 (100.0)	98.3 (79.2)
$\langle I/\sigma(I) \rangle$	15.18 (3.02)	9.00 (1.14)	15.40 (1.02)	22.43 (3.22)
$R_{sym}(I)$	0.135 (0.964)	0.161 (1.541)	0.182 (3.355)	0.145 (1.211)
$CC_{1/2}$	0.998 (0.781)	0.998 (0.516)	0.999 (0.555)	0.999 (0.889)
**Refinement**				
Nr Reflections work/free sets	47756 / 1436	40022 / 2042	52325 / 1532	47789 / 1916
R_{work} / R_{free}	0.173 / 0.198	0.175 / 0.220	0.186 / 0.211	0.1859/0.2103
A.u. Components				
Nr Protein atoms	4806	4806	4712	4877
Ligands/ions	2x Fe^{2+}	2x Fe^{2+} / 2x GA	2x Fe^{2+} / 1x NOG	2x Fe^{2+} / 2x SA
Water	519	287	272	527
B-factors ($Å^2$)				
Protein	35.20	44.61	52.45	45.30
Ligand/ion	34.39	80.76	87.79	75.02
Water	38.43	49.51	56.03	53.06
R.m.s deviations				
Bond lengths (Å)	0.003	0.008	0.008	0.011
Bond angles (°)	0.736	0.923	0.940	1.233

Supplementary Table 2:Ions used for identification and quantification of compounds by LC-MS

Compound	Retention time	Ions	*m/z*
Glutaric semialdehyde	4.3	$[M-H]^-$	115.0401
Glutaric acid	4.9	$[M-H]^-$	131.0350
Succinic acid	5.4	$[M-H]^-$	117.0193
L-2-Hydroxyglutaric acid	10.0	$[M-H]^-$	147.0299
Piperideine dimer[1]	10.5	$[M+H]^+$	167.1543
5-Aminopentanal[1]	11.6	$[M+H]^+$; $[M-H_2O+H]^+$	102.0913; 84.0808
α-Ketoglutarate	12.0	$[M-H]^-$	145.0425
5-Aminovalerate	12.5	$[M+H]^+$	118.0862
Glutamic acid	13.7	$[M+H]^+$	148.0604
Piperideine monomer[1]	15.3	$[M+H]^+$; $[2M+H]^+$	84.0808; 167.1543
Cadaverine	16.1	$[M+H]^+$	103.1230

High resolution ESI-MS (direct injection) and LC-MS measurements on an Orbitrap MS were used for compound identification. Quantification was performed in SIM mode on a quadrupole LC-MS (see Materials and Methods for details).

[1]: Signal intensities of ions at *m/z* 84.1 (RT=11.6 and 15.3 min), 102.1 (RT=11.6 min) and 167.2 (RT=10.5 min) corresponding to piperideine monomer, aminopentanal and piperideine dimer, respectively, were summed up for 5-aminopentanal quantification.

Supplementary Table 3: Labelling pattern and total quantification of metabolites measured via HPLC/MS of *E. coli* WT, Δ*csiD* and Δ*gabT*

Compound		Concentration[3]	Intermediate distribution (%) for labeling atoms incorporated (mass shift [M+X] listed)								
		µM	X=0	X=1	X=2	X=3	X=4	X=5	X=6	X=7	X=8
Cadaverine	*WT*	1829±56	2.0±0.6	-	-	-	-	-	4.8±0.1	93.2±0.5	-
	Δ*csiD*	1233±54	1.9±0.4	-	-	-	-	-	4.7±0.1	93.4±0.4	-
	Δ*gabT*	768±53	4.9±0.4	-	-	-	-	-	4,8±0.2	90.3±0.4	-
Piperideine /	*WT*	753±112	-	-	-	-	-	4.9±0.6	95.1±0.6	-	-
Aminopentanal[1]	Δ*csiD*	708±90	-	-	-	-	-	5.2±0.9	94.8±0.9	-	-
	Δ*gabT*	535±76	-	-	-	-	-	4.6±0.7	95.4±0.7	-	-
Aminovalerate[2]	*WT*	n.d.	n.d.	-	-	-	-	-	-	-	-
	Δ*csiD*	n.d.	n.d.	-	-	-	-	-	-	-	-
	Δ*gabT*	637±68	n.d.	-	-	-	-	4.8±0.4	95.2±0.4	-	-
Glutaric acid	*WT*	180±16	9.1±1.5	-	-	-	4,6±0.3	86.3±1.4	-	-	-
	Δ*csiD*	4994±372	2.7±0.2	-	-	-	4.7±0.1	92,5±0.2	-	-	-
	Δ*gabT*	357±36	9.5±1.5	-	-	-	4.6±0.3	85.9±1.6	-	-	-
Succinic acid	*WT*	598±101	95.4±1.5	-	3.4±0.9	-	1.8±0.5	-	-	-	-
	Δ*csiD*	498±31	100	-	-	-	-	-	-	-	-
	Δ*gabT*	782±48	93.6±1.0	-	3.8±0.6	-	2.7±0.6	-	-	-	-

(-): not detectable. Grey squares marked the mass shifts expected due to the described pathway. Mass shifts of [M+X-1] found at an abundancy of ~ 5% can be attributed to isotopic impurity of labeled lysine used (composed of 95% [M+8] and 5% [M+7]), in accordance with the manufacturer´s reported purity of the isotope-labelled lysine. Succinic acid semialdehyde was not detectable.

[1]Due to the spontaneous conversion of 5-Aminopentanal to Piperideine, monomer and dimer under cell cultivation[5], sample preparation, and measurement conditions, we used summarized concentrations of these compounds.

[2]Aminovalerate (RT=12.33 min) cannot be completely separated from valine (RT=12.52 min). Due to the same sum formula and its low abundancy the concentration, the amount of non-labeled aminovalerate and hence the total amount of aminovalerate could not be determined (n.d.).

[3] Mean ± s.d.; n ≥ 3

Supplementary Table 4: List of primers used in the study

Name	Sequence	Description
**MS161**	CCCGATAAAACGGGGCAGATAA	
**MS162**	ATAGTCCTGGCCTGAATCGACA	
**MS170**	TGTTGCTTTTGATCACAATAAG	
**MS171**	TATGAGATGTAGGGTGACATGG	
**MS177**	[Btn]CTTTTGTGCGCATTTT	
**MS178**	CTCATTTCGTAGCCATAA	
**SP04**	TCCCCATCGGTGATGTC	fw sequencing primer for pET28a
**SP10**	CTAGTTATTGCTCAGCGG	rv sequencing primer for pET28a
**SK80**	aaaaaCATATGAATGCACTGACCGCCGTACA	fw primer for csiD (*E. coli*) gene amplification + NdoI site
**SK81**	tttttCTCGAGTTACTGATGCGTCTGGTAGT	rv primer for csiD (*E. coli*) gene amplification + XhoI site
**SK99**	aaaaaCATATGAACAGCAATAAAGAGTTAATGCAG	fw primer for gabT (*E. coli*) gene amplification + NdoI site
**SK100**	tttttCTCGAGCTACTGCTTCGCCTCATCAAA	rv primer for gabT (*E. coli*) gene amplification + XhoI site
**SK101**	aaaaaCATATGAAACTTAACGACAGTAACTTATTCCG	fw primer for gabD (*E. coli*) gene amplification + NdoI site
**SK102**	tttttCTCGAGTTAAAGACCGATGCACATATATTT	rv primer for gabD (*E. coli*) gene amplification + XhoI site
**SK103**	aaaaaCATATGACCATTACGTCTCTGGATGGCT	fw primer for csiR (*E. coli*) gene amplification + NdoI site
**SK104**	tttttCTCGAGTTAATTGCCAGCCATCGCCT	rv primer for csiR (*E. coli*) gene amplification + XhoI site
**SK121**	aaaaaCATATGACCATTACTCCGGCAACTCA	fw primer for sad (yneI) (*E. coli*) gene amplification + NdoI site
**SK122**	tttttCTCGAGTCAGATCCGGTCTTTCCACA	rv primer for sad (yneI) (*E. coli*) gene amplification + XhoI site
**SK123**	aaaaaCATATGAGCAACAATGAATTCCATCAGC	fw primer for puuE (*E. coli*) gene amplification + NdoI site
**SK124**	tttttCTCGAGTTAATCGCTCAGCGCATCCT	rv primer for puuE (*E. coli*) gene amplification + XhoI site
**SK137**	aaaaaCCATGGGGATGTATGATT	fw primer for lhgO (*E. coli*) gene amplification + NcoI site
**SK138**	tttttCTCGAGTTGATTAAATGCGGCGTGT	rv primer for lhgO (*E. coli*) gene amplification + XhoI site
**MS205**	cgacGGATCCTCAGTGGTGGTGGTGGTG	rv primer for lhgO (*E. coli*) gene amplification + BamHI site, *Δlhgo* complementation

References

1 Struys, E. A., Jansen, E. E., Verhoeven, N. M. & Jakobs, C. Measurement of urinary D- and L-2-hydroxyglutarate enantiomers by stable-isotope-dilution liquid chromatography-tandem mass spectrometry after derivatization with diacetyl-L-tartaric anhydride. *Clin Chem* **50**, 1391-1395, doi:10.1373/clinchem.2004.033399 (2004).

2 O'Leary, N. A. *et al.* Reference sequence (RefSeq) database at NCBI: current status, taxonomic expansion, and functional annotation. *Nucleic acids research* **44**, D733-745, doi:10.1093/nar/gkv1189 (2016).

3 Xu, W. *et al.* Oncometabolite 2-hydroxyglutarate is a competitive inhibitor of alpha-ketoglutarate-dependent dioxygenases. *Cancer Cell* **19**, 17-30, doi:10.1016/j.ccr.2010.12.014 (2011).

4 Das, R., Laederach, A., Pearlman, S. M., Herschlag, D. & Altman, R. B. SAFA: semi-automated footprinting analysis software for high-throughput quantification of nucleic acid footprinting experiments. *RNA* **11**, 344-354, doi:10.1261/rna.7214405 (2005).

5 Sato, H., Uchiyama, M., Saito, K. & Yamazaki, M. The Energetic Viability of Delta(1)-Piperideine Dimerization in Lysine-derived Alkaloid Biosynthesis. *Metabolites* **8**, doi:10.3390/metabo8030048 (2018).
